# Supplementary figures and images for: Assembly of infectious Kaposi’s sarcoma-associated herpesvirus progeny requires formation of a pORF19 pentamer
Source: PLoS Biol. 2021 Nov 4;19(11):e3001423. doi: 10.1371/journal.pbio.3001423 (PMC8568140; doi:10.1371/journal.pbio.3001423)

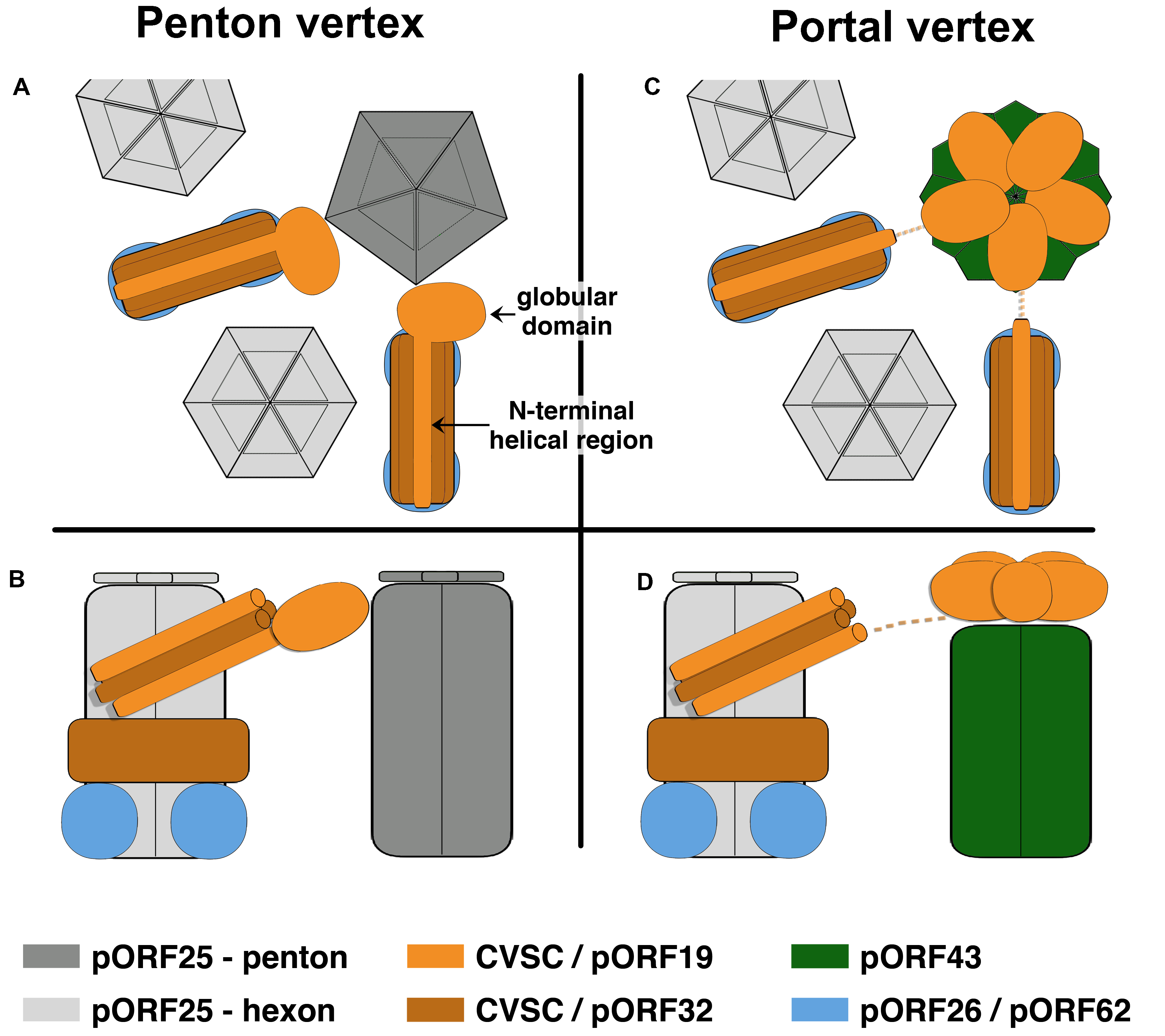

Supplement: S1 Fig — Schematic view of the penton vertex (A, B) and the portal vertex (C, D), viewed from above (top) and from the side (bottom) illustrating the main differences. For clarity, the constituents of CVSC and PVAT are colored in orange with different shades depicting pORF19 and pORF32, respectively, and the SCP pORF65 is shown as triangle in light and dark gray on hexons and pentons, respectively. CVSC, capsid vertex–specific component; KSHV, Kaposi’s sarcoma-associated herpesvirus; PVAT, portal vertex–associated tegument; SCP, small capsid protein. (TIF) [file pbio.3001423.s001.tif]

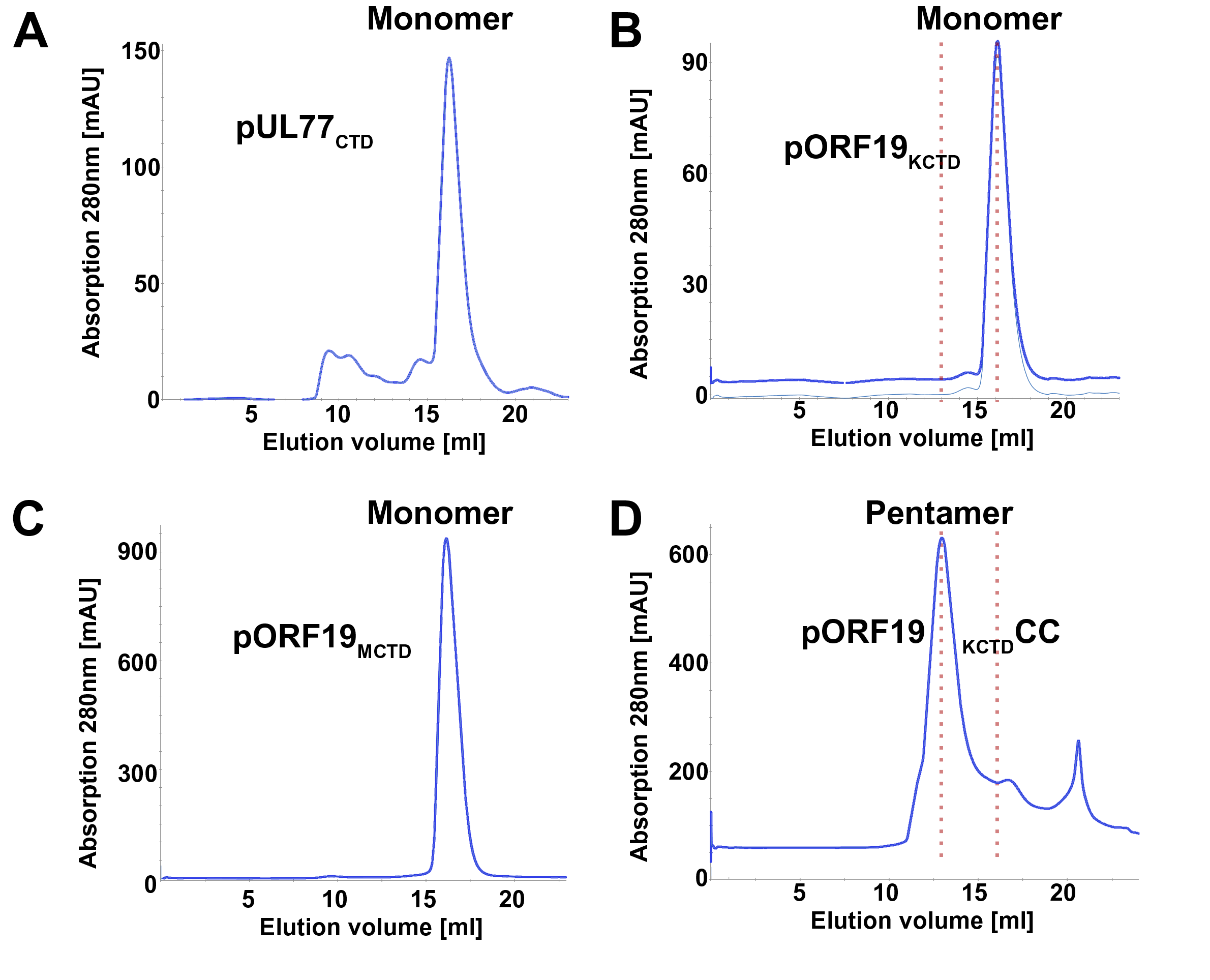

Supplement: S2 Fig — (A–D) Elution profiles from a Superdex 200 Increase 10/300 GL size exclusion column obtained upon purification of individual CTDs from HCMV pUL77 (A), KSHV pORF19 (B), and MuHV-68 pORF19 (C). In all cases, the majority of the protein elutes at a volume presumably corresponding to a monomer. (D) Elution profile of oxidized pORF19KCTDCC analyzed by SEC aligned to the profile of the non-oxidized wt pORF19KCTD. The vertical red dotted lines mark the elution volume of the oxidized pORF19KCTDCC and the non-oxidized wt pORF19KCTD (B). The oxidized protein elutes at an earlier volume, underlining the difference in oligomeric state. The underlying data for all chromatograms can be found in S3 Data. CTD, carboxyl-terminal domain; HCMV, human cytomegalovirus; KSHV, Kaposi’s sarcoma-associated herpesvirus; MuHV-68, murid gammaherpesvirus 68; SEC, size exclusion chromatography; wt, wild-type. (TIF) [file pbio.3001423.s002.tif]

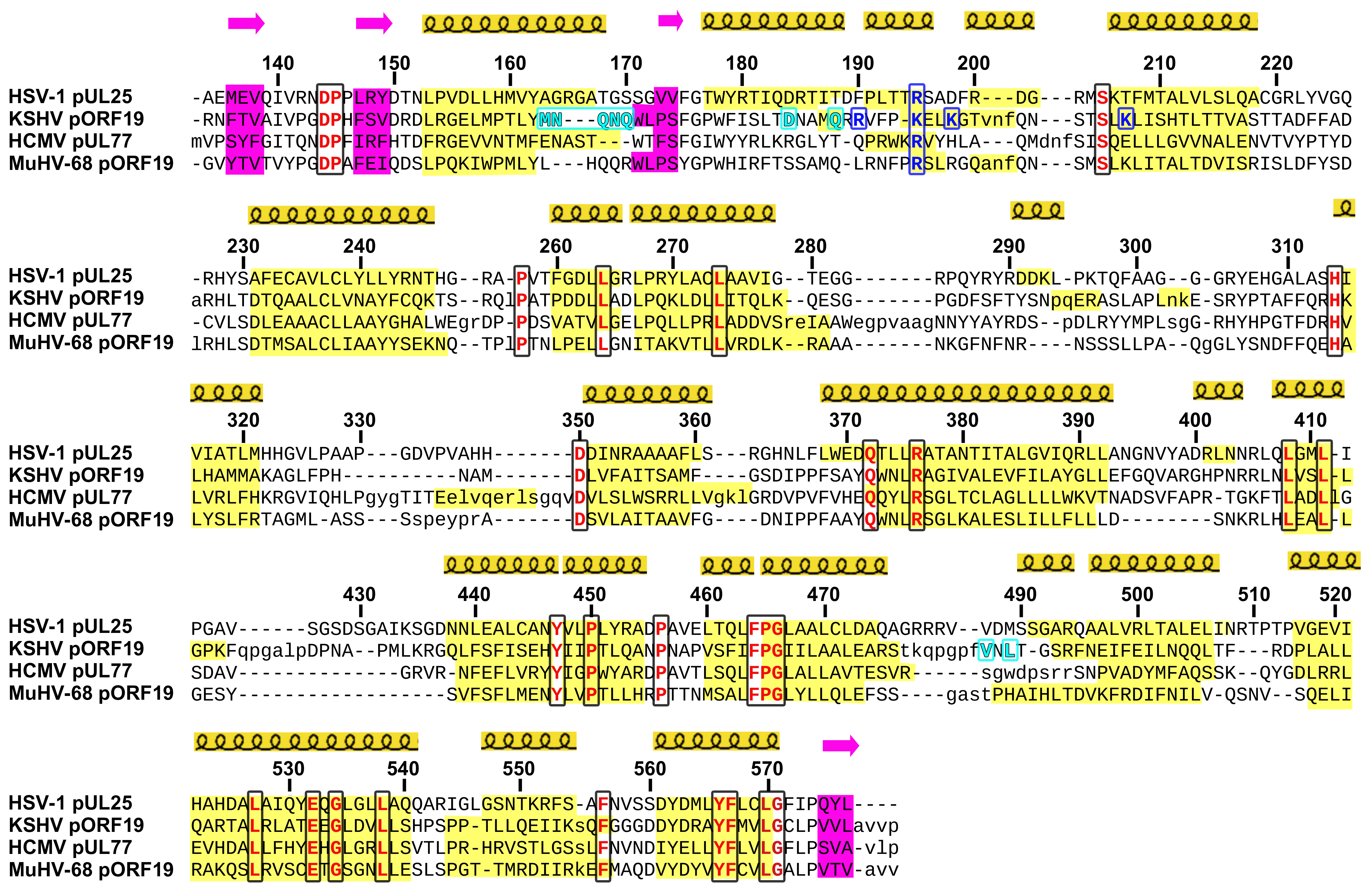

Supplement: S3 Fig — Structural alignment of the globular domains of HSV-1 pUL25 (PDB 2F5U), HCMV pUL77, KSHV pORF19, and MuHV-68 pORF19, obtained via a pairwise comparison with the Dali server [37]. Magenta or yellow background color indicates the SSE of the individual proteins as determined by the ENDscript server [81]; numbering and SSEs of HSV-1 pUL25 are shown above the alignment. Residues in red within black framed boxes are conserved across all 4 herpesvirus orthologs. Lowercase letters denote insertions relative to HSV-1 pUL25. Residues mutated in this study to block pentamerization are colored and framed in cyan and the positively charged residues in the funnel region of the pentameric pORF19KCTD in blue. HCMV, human cytomegalovirus; HSV, herpes simplex virus; KSHV, Kaposi’s sarcoma-associated herpesvirus; MuHV-68, murid gammaherpesvirus 68; SSE, secondary structure element. (TIF) [file pbio.3001423.s003.tif]

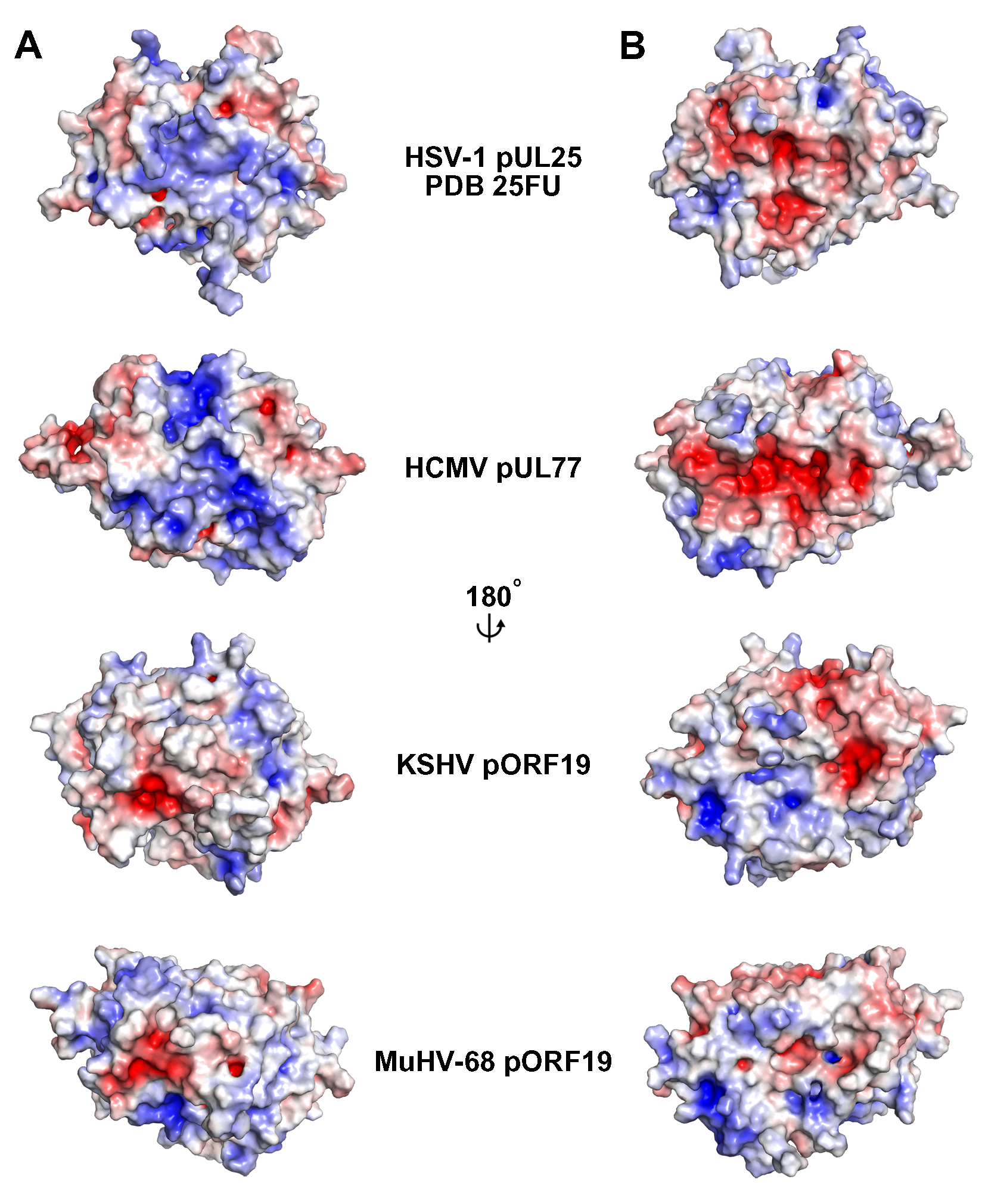

Supplement: S4 Fig — (A+B) View on the face of pUL25 previously described to contain a large number basic patches representing positive charges (left panel, PDB 2F5U) and the opposite face (right panel) compared with the charge distribution on the surface of pUL25 orthologs in the same orientation). The electrostatic potential is represented and calculated as for Fig 3. CTD, carboxyl-terminal domain. (TIF) [file pbio.3001423.s004.tif]

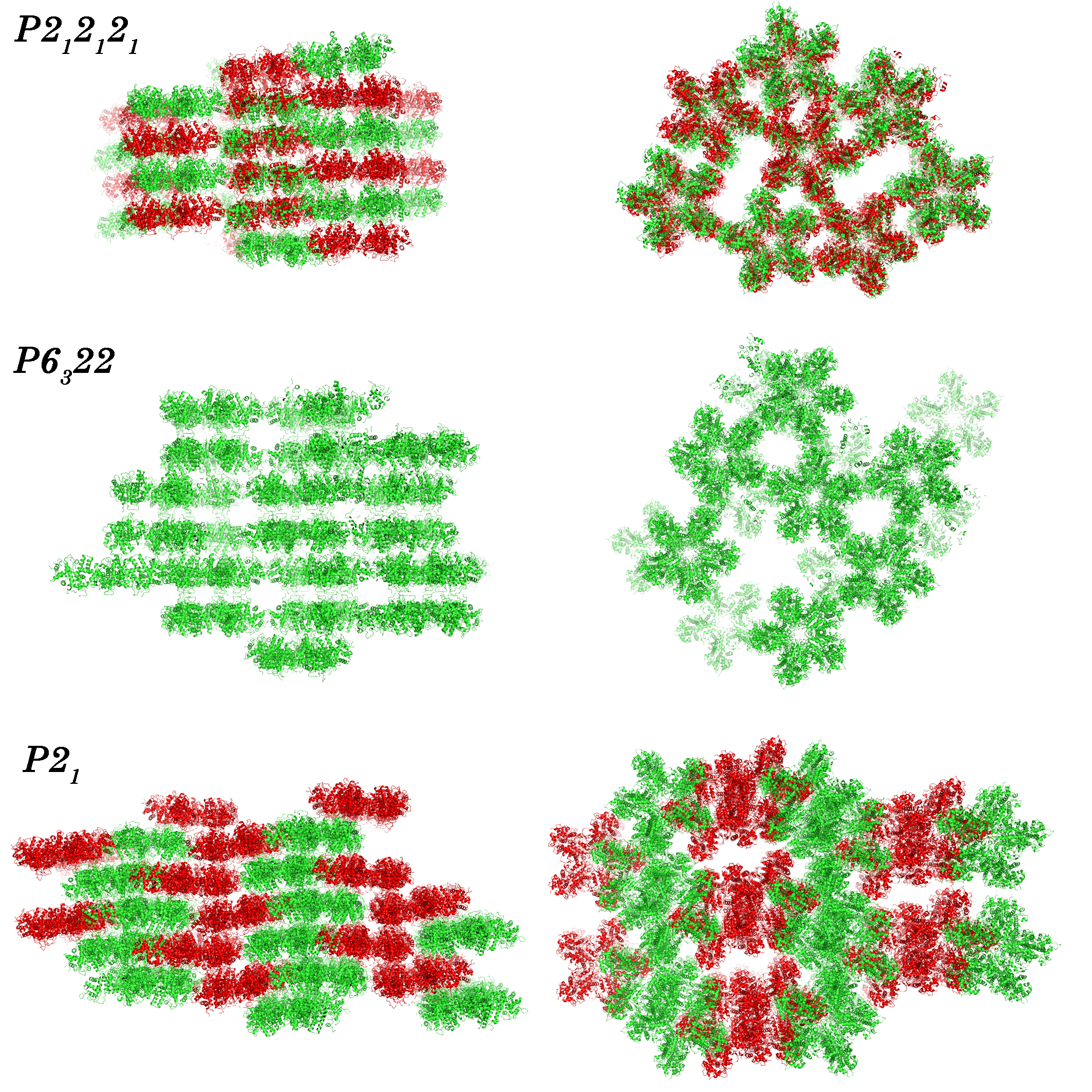

Supplement: S5 Fig — Crystalline arrangement of the pentameric pORF19KCTD rings in the P212121 space group (top panel; 2 rings per AU), the P6322 space group (middle panel; 1 ring per AU) and the P21 space group (bottom panel; 2 rings per AU) in the side view (left) and top view (right). The first pentameric ring in each AU is colored green, the second one (spacegroups P212121 and P21) is colored red to illustrate the respective crystal packing environment. In all cases, the rings interact laterally to form layers that are densely stacked; however, the type of lateral interactions differs between individual crystal lattices, suggesting that a pentamer is the functional assembly unit. AU, asymmetric unit; CTD, carboxyl-terminal domain. (TIF) [file pbio.3001423.s005.tif]

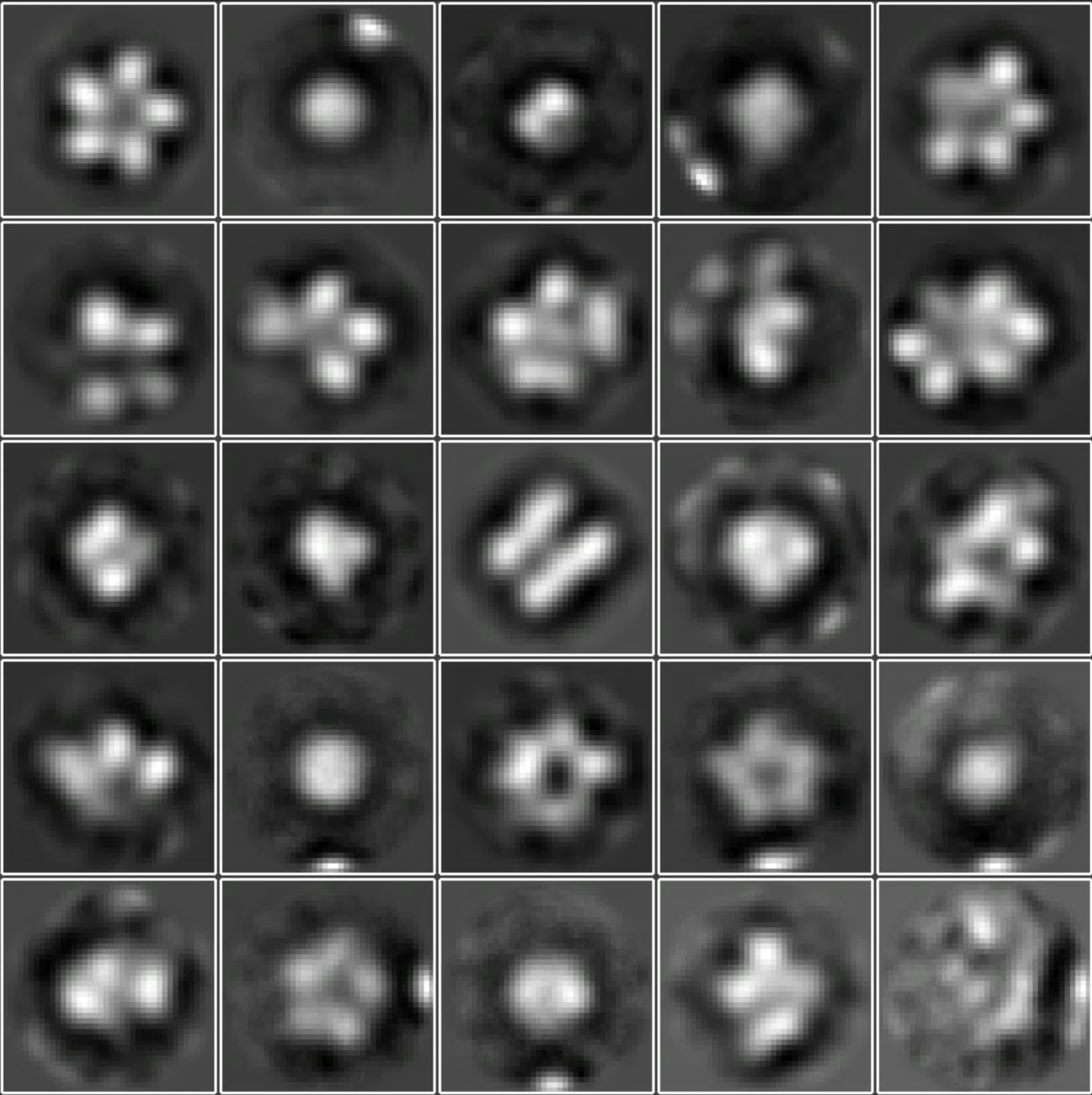

Supplement: S6 Fig — Negative stain EM images were used for single particle analysis and obtained 2D class averages are shown. CTD, carboxyl-terminal domain; EM, electron microscopy. (TIF) [file pbio.3001423.s006.tif]
