# Supplementary material for: Assembly of infectious Kaposi’s sarcoma-associated herpesvirus progeny requires formation of a pORF19 pentamer
Source: PLoS Biol. 2021 Nov 4;19(11):e3001423. doi: 10.1371/journal.pbio.3001423 (PMC8568140; doi:10.1371/journal.pbio.3001423)
Supplement: S1 Table — (DOCX) [file pbio.3001423.s007.docx]

|  | **HSV-1** | **HCMV** | **KSHV / MuHV-68** |  |
| --- | --- | --- | --- | --- |
| CVSC component 1 | pUL25 | pUL77 | pORF19 |  |
| CVSC component 2 | pUL17 | pUL93 | pORF32 | |
| Major capsid protein (MCP) | pUL19 | pUL86 | pORF25 | |
| Portal protein | pUL6 | pUL104 | pORF43 | |
| Small capsid protein (SCP) | pUL35 | pUL48A | pORF65 | |
| Triplex dimer protein | pUL18 | pUL85 | pORF26 | |

**S1 Table. Nomenclature of proteins important for capsid assembly across herpesviruses.**
