# Supplementary material for: Assembly of infectious Kaposi’s sarcoma-associated herpesvirus progeny requires formation of a pORF19 pentamer
Source: PLoS Biol. 2021 Nov 4;19(11):e3001423. doi: 10.1371/journal.pbio.3001423 (PMC8568140; doi:10.1371/journal.pbio.3001423)
Supplement: S2 Table — (DOCX) [file pbio.3001423.s008.docx]

|  | **pUL77_CTD_** | **pORF19_KCTD_** | **pORF19_MCTD_** |
| --- | --- | --- | --- |
| **Data collection** |  |  |  |
| Space group | *P 4_1_ 2 2* | *P 2_1_2_1_2_1_* | *P 6_1_* |
| Molecules per AU | 1 | 10 | 1 |
| Cell dimensions |  |  |  |
| *a, b, c* (Å) | 163.91 163.91 50.97 | 95.39 238.56 263.87 | 55.16 55.16 224.90 |
| *α, β, γ* (°) | 90.00 90.00 90.00 | 90.00 90.00 90.00 | 90.00 90.00 120.00 |
| Resolution (Å) | 48.66-1.90 (2.01-1.90) | 49.66-2.42 (2.56-2.42) | 47.77-1.90 (2.01-1.90) |
| Rmeas | 0.09 (1.505) | 0.179 (3.952) | 0.131 (1.574) |
| Completeness (%) | 99.5 (97.4) | 99.1 (94.5) | 99.7 (98.2) |
| Redundancy | 8.6 (8.5) | 13.3 (13.3) | 15.8 (15.4) |
| I/ σ^I^ | 16.7 (1.5) | 10.6 (0.8) | 15.3 (1.3) |
| CC_1/2_ | 99.9 (65.9) | 99.8 (49.4) | 99.9 (84.9) |
|  |  |  |  |
| **Refinement** |  |  |  |
| No. reflections | 55182 | 174243 | 30304 |
| Rwork/ Rfree | 0.1824 / 0.1934 | 0.207 / 0.235 | 0.194 / 0.242 |
|  |  |  |  |
| **No. of atoms** |  |  |  |
| Macromolecules | 3411 | 31860 | 3041 |
| Ligand | 0 | 0 | 0 |
| Water | 184 | 1980 | 167 |
| Residues per AU | 425 | 4056 | 385 |
|  |  |  |  |
| **B-factors** |  |  |  |
| Average B-factor (Å^2^) | 41.68 | 60.87 | 42.48 |
|  |  |  |  |
| **Ramachandran Statistics** |  |  |  |
| Favored (%) | 97.6 | 98.4 | 98.4 |
| Allowed (%) | 2.4 | 1.6 | 1.6 |
| Outliers (%) | 0 | 0 | 0 |
|  |  |  |  |
| **R.m.s. deviations** |  |  |  |
| Bond length (Å) | 0.010 | 0.006 | 0.010 |
| Bond angles (°) | 0.88 | 0.81 | 1.00 |

**S2 Table. Diffraction data collection and refinement statistics.**

^1^ Values in parentheses correspond to the highest resolution shell. rms, root-mean-square.

^2^ Ramachandran statistics were calculated with MolProbity.
