# Supplementary material for: Assembly of infectious Kaposi’s sarcoma-associated herpesvirus progeny requires formation of a pORF19 pentamer
Source: PLoS Biol. 2021 Nov 4;19(11):e3001423. doi: 10.1371/journal.pbio.3001423 (PMC8568140; doi:10.1371/journal.pbio.3001423)
Supplement: S3 Table — (DOCX) [file pbio.3001423.s009.docx]

| **pUL77_CTD_** | **pORF19_KCTD_** | **pORF19_MCTD_** |  |
| --- | --- | --- | --- |
| 400 - 401 | 320 - 329 | 345 – 350 |  |
| 472 - 492 | 400 - 407 | 366 – 383 | |
| 544 - 562 | 454 - 456 | 450 - 452 | |

**S3 Table. Missing loops in the ortholog structures.**
