# Supplementary material for: Assembly of infectious Kaposi’s sarcoma-associated herpesvirus progeny requires formation of a pORF19 pentamer
Source: PLoS Biol. 2021 Nov 4;19(11):e3001423. doi: 10.1371/journal.pbio.3001423 (PMC8568140; doi:10.1371/journal.pbio.3001423)
Supplement: S4 Table — Statistics from a pairwise analysis of listed pUL25 orthologs using the DALI server [37]. For reference, Z scores below 2 are meaningless, whereas values of around 50 are obtained when structures of the same protein from 2 different crystal forms are compared (serving as reference for proteins of roughly the same size). rmsd is the root mean square deviation between Cα atoms (in Å). The third line in each box is “N/NT”, where the number of aligned residues (N) is compared to the total residues in the alignment (NT). “%id” indicates % amino acid identity after the alignment. (DOCX) [file pbio.3001423.s010.docx]

|  | ***HSV-1 pUL25***  ***(PDB 2F5U)*** | ***HCMV pUL77*** | ***KSHV pORF19*** | ***MuHV-68 pORF19*** |
| --- | --- | --- | --- | --- |
| ***HSV-1 pUL25***  ***(PDB 2F5U)*** |  | **z=43.3**  **rmsd=2.4**  **375/423**  **25 %id** | **z=45.8**  **rmsd=2.5**  **381/403**  **21 %id** | **z=42.9**  **rmsd=2.5**  **363/385**  **20 %id** |
| ***HCMV pUL77*** |  |  | **z=45.8**  **rmsd=2.2A**  **373/403**  **26 %id** | **z=43.5**  **rmsd=2.5**  **368/385**  **23 %id** |
| ***KSHV pORF19*** |  |  |  | **z=54.2**  **rmsd=1.6**  **373/385**  **42 %id** |
| ***MuHV-68 pORF19*** |  |  |  |  |

**S4 Table. Structural similarity across orthologs.** Statistics from a pairwise analysis of listed pUL25 orthologs using the DALI server (Holm and Rosenström, 2010). For reference, Z scores below 2 are meaningless, whereas values of around 50 are obtained when structures of the same protein from two different crystal forms are compared (serving as reference for proteins of roughly the same size). rmsd is the root mean square deviation between Cα atoms (in Å). The third line in each box is “N/N^T”^, where the number of aligned residues (N) is compared to the total residues in the alignment (N^T^). “%id” indicates % amino acid identity after the alignment.
