# Supplementary material for: Assembly of infectious Kaposi’s sarcoma-associated herpesvirus progeny requires formation of a pORF19 pentamer
Source: PLoS Biol. 2021 Nov 4;19(11):e3001423. doi: 10.1371/journal.pbio.3001423 (PMC8568140; doi:10.1371/journal.pbio.3001423)
Supplement: S5 Table — Protein concentration was 127 μM in all cases. Percentages of “monomer” and “oligomer” were calculated by integration of the monomer and the oligomer peaks as indicated in the c(s) distributions obtained from the sedimentation profiles in Fig 4A, right panel. Since monomer and oligomer co-sediment in the faster moving boundary, the concentration of the oligomer is thereby overestimated and should not be taken as an absolute value, but as a measure for the ability of the different conditions to promote pORF19KCTD oligomerization. CTD, carboxyl-terminal domain. (DOCX) [file pbio.3001423.s011.docx]

| **[LiAc]** | **[LiCl]** | **[MgAc_2_]** | **[NaAc]** | **[NaCl]** | **Monomer (in %)** | **Oligomer (in %)** |  |
| --- | --- | --- | --- | --- | --- | --- | --- |
| 0.15 M **^a^** |  |  |  |  | 100 | 0 |  |
| 0.3 M **^a^** |  |  |  |  | 100 | 0 | |
| 0.5 M **^a^** |  |  |  |  | 62.1 | 37.9 | |
|  | 0.5 M **^b^** |  |  |  | 96.9 | 3.1 | |
|  |  | 0.15 M **^a^** |  |  | 97.2 | 2.8 | |
|  |  | 0.3 M **^a^** |  |  | 96.6 | 3.4 | |
|  |  | 0.5 M **^a^** |  |  | 94.8 | 5.2 | |
|  |  |  | 0.5 M **^b^** |  | 99.9 | 0.1 | |
|  |  |  |  | 0.3 M **^b^** | 100 | 0 | |
|  |  |  |  | 0.5 M **^b^** | 99.3 | 0.7 | |
|  |  |  |  | 1 M **^b^** | 97.8 | 2.2 | |

**S5 Table. Efficiency of pORF19_KCTD_ pentamerization in vitro.** Protein concentration was 127 μM in all cases. Percentages of ‘monomer’ and ‘oligomer’ were calculated by integration of the monomer and the oligomer peaks as indicated in the c(s) distributions obtained from the sedimentation profiles in Fig. 4A, right panel. Since monomer and oligomer co-sediment in the faster moving boundary, the concentration of the oligomer is thereby overestimated and should not be taken as an absolute value, but as a measure for the ability of the different conditions to promote pORF19_KCTD_ oligomerization.

^a^ 50 mM HEPES pH 7.5

^b^ 100 mM HEPES pH 7.5
