# Supplementary material for: Assembly of infectious Kaposi’s sarcoma-associated herpesvirus progeny requires formation of a pORF19 pentamer
Source: PLoS Biol. 2021 Nov 4;19(11):e3001423. doi: 10.1371/journal.pbio.3001423 (PMC8568140; doi:10.1371/journal.pbio.3001423)
Supplement: S6 Table — (DOCX) [file pbio.3001423.s012.docx]

| **Primer name** |  |
| --- | --- |
| PNHV1 | TCTAGAAATAATTTTGTTTAACTTTAAGAAGGAGATATACCATGGCCAGCCACCGTAACTTCAC |
| PNHV2 | AGTGCGGCCGCAGGAACGACCGCGAGGACCA |
| PNHV134 | CATGGCACGGCGCGTCTTCCCTAAAG |
| PNHV135 | GCGTTTGCCGTGAGGCTGATGAACC |
| PNHV138 | GGTGGCAGCTGGTTGCCATCCTTTGGG |
| PNHV139 | GTACAGGGTGGGCATGAGCTCG |
| PNHV136 | ATTACACAGGCAGTAGGTTTAATGAGATATTC |
| PNHV137 | TCCAAAATGGTCCGGGTTGTTTGTGGG |
| PNHV3 | CCATGGTACCTAGCTACTTTGGAATCACGCAGAACG |
| PNHV4 | TTGCGGCCGCAGGCAACACCGCCACGCTCGGAA |
| PNHV196 | TAATTTTGTTTAACTTTAAGAAGGAGATATACCATGGTGAGCAAGGGCGAGG |
| PNHV197 | AGGTGTGCAGCAGGCTTCCCATGCCCTGAAAATAAAGATTCTCCTTGTACAGCTCGTCCATGCC |
| ORF19 C KO Fwd | GACAACATAGACATGCCCCAAAGCCGTTCCCATGAGCCTCCCTTAGTGACATCCCCCCCGCAGGACGCATCGTGGCCGGATCTC |
| ORF19 C KO Rev | GGTTATCTTCCGGCTCCGTCCGAAGCGCAGCTGGCCTACCTTGGTGCGTTTAACAACAACGCGGTGTGACCACGTCGTGGAATGC |
| BAC16 D Fwd | GAACCAGAATCAGTGGTTGCCATCCTTTGGGCCGTGG  TTCATCAGCCTCACGGCAAACGCCATGGCACGGCGCGTCTTCCCTAAAGAACGACGCATCGTGGCCGGATCTC |
| BAC16 D Rev | GAAAGTTAACAGTCCCCTTTAGTTCTTTAGGGAAGACGC  GCCGTGCCATGGCGTTTGCCGTGAGGCTGATGAACCACGGCCCGTGACCACGTCGTGGAATGC |
| BAC16 V Fwd | CTGGCCGCCCTCGAGGCGCGATCGACCGTGTCCCACA  AACAACCCGGACCATTTTGGAATTACACAGGCAGTAGGTTTAATGACGCATCGTGGCCGGATCTC |
| BAC16 V Rev | GTTCAGGATTTCGAATATCTCATTAAACCTACTGCCTGTG  TAATTCCAAAATGGTCCGGGTTGTTTGTGGGACACGGTCGATGTGACCACGTCGTGGAATGC |
| BAC16 Y Fwd | CCCGGGGACCCTCACTTCTCCGTTGACAGGGACCTGC  GCGGCGAGCTCATGCCCACCCTGTACGGTGGCAGCTGGTTGCCATCCTTTGGGCCGTGGTTCATCAGCGACGCATCGTGGCCGGATCTC |
| BAC16 Y Rev | CTGCATGGCGTTGTCCGTGAGGCTGATGAACCACGGCC  CAAAGGATGGCAACCAGCTGCCACCGTACAGGGTGGGCATGAGCTCGCCGCGCAGGTCCCTGTCAACGTGACCACGTCGTGGAATGC |

**S6 Table. List of oligonucleotides used in this study.**
