# Supplementary material for: Assembly of infectious Kaposi’s sarcoma-associated herpesvirus progeny requires formation of a pORF19 pentamer
Source: PLoS Biol. 2021 Nov 4;19(11):e3001423. doi: 10.1371/journal.pbio.3001423 (PMC8568140; doi:10.1371/journal.pbio.3001423)

Blot Fig. 5A, left panel, α-pORF26

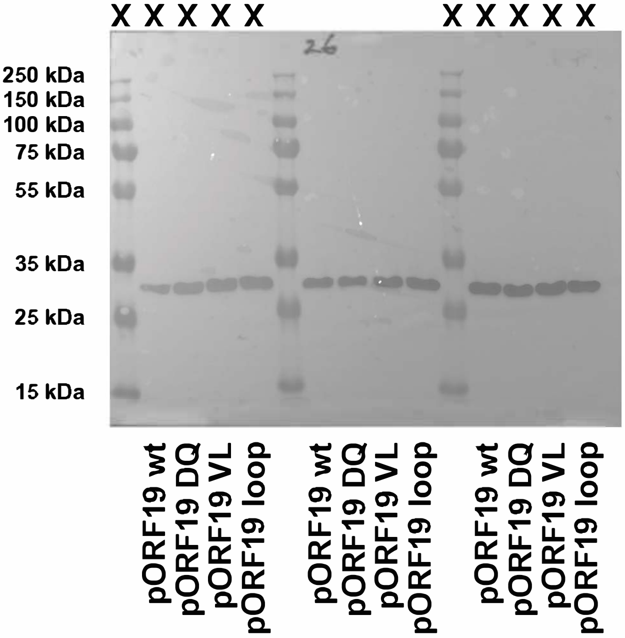

Blot Fig. 5A, middle panel, α-V5 (pORF25)

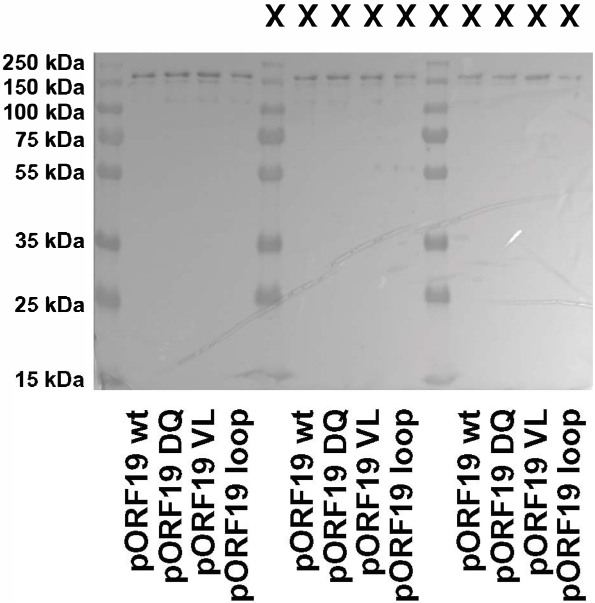

Blot Fig. 5A, right panel, α-GFP (pORF19)

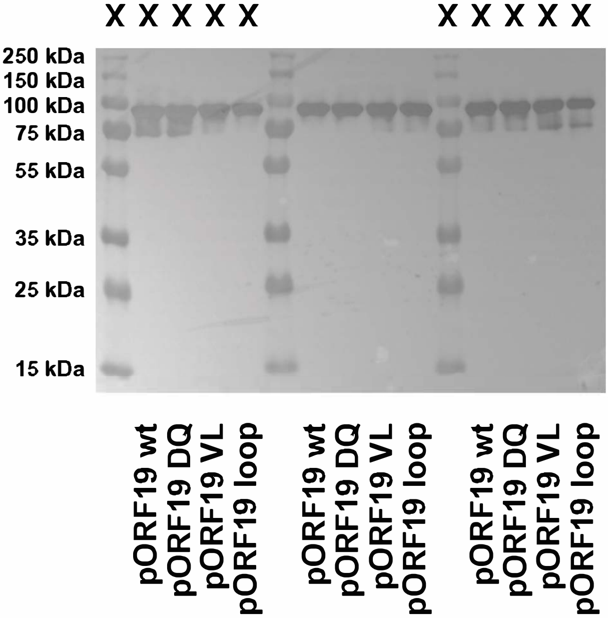

Blot Fig. 6A, top / bottom panel, α-LANA / α-β-Actin

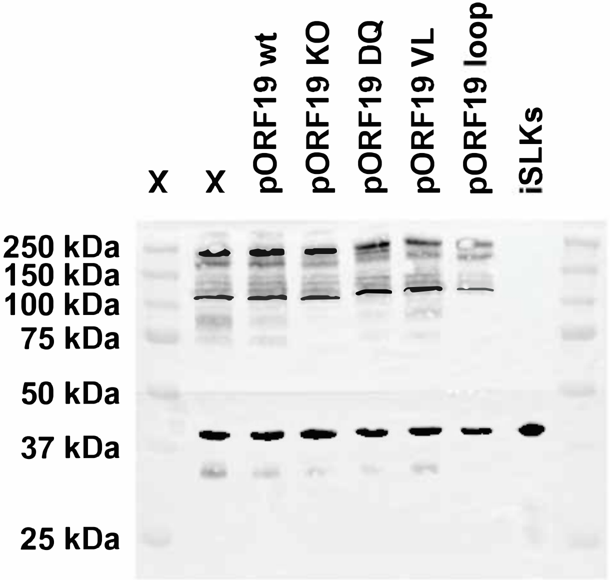

Blot Fig. 6A, middle panels, α-pORF45 / α-K-bZIP

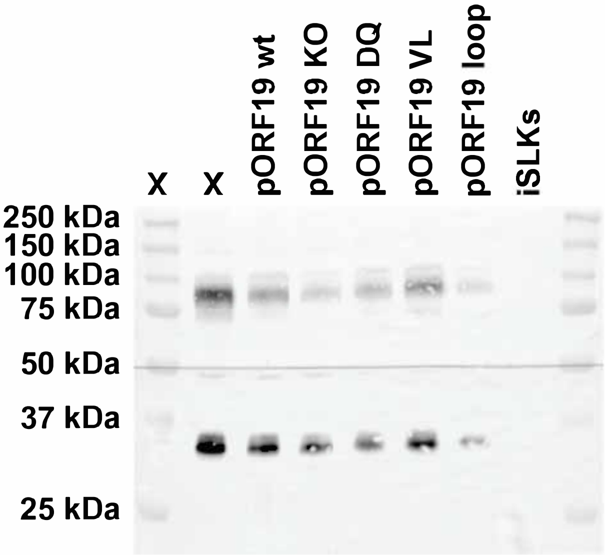

Blot Fig. 6C, top panel, α-gH

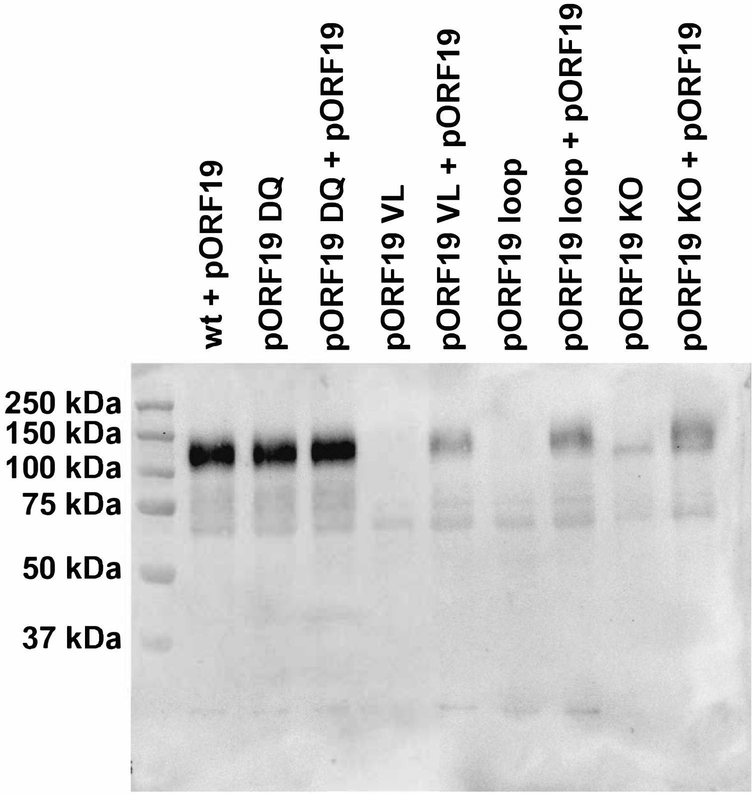

Blot Fig. 6C, middle panel, α-pORF45

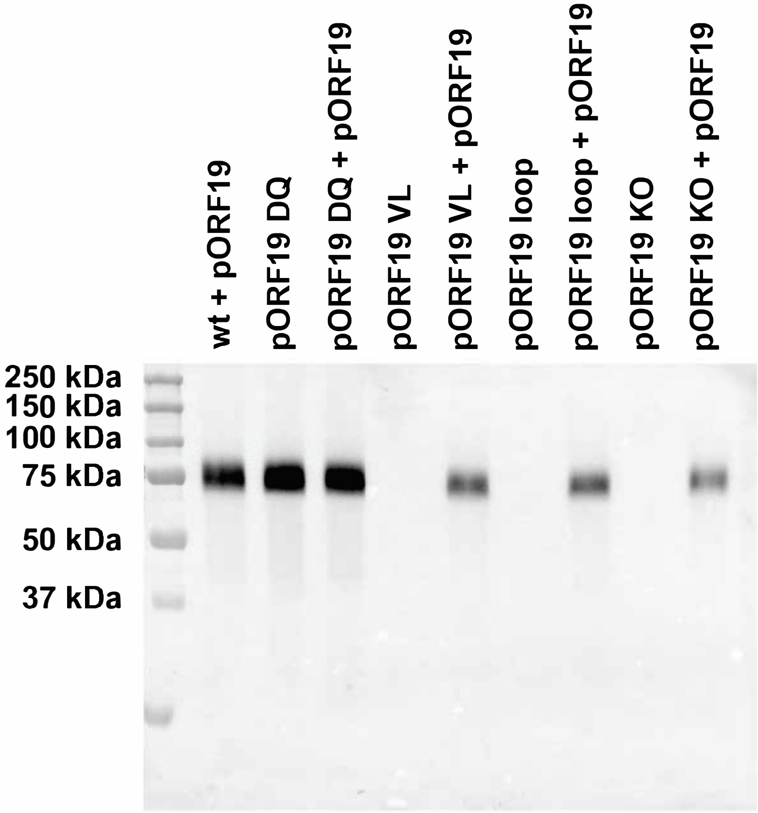

Blot Fig. 6C, bottom panel, α-pORF26

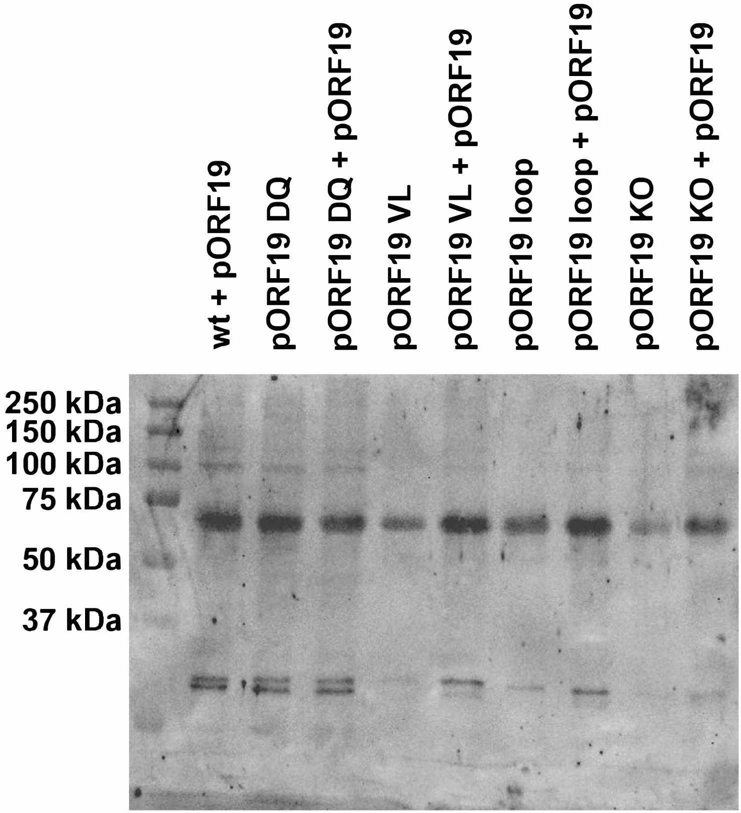

Supplement: S1 Raw Images — The “X” designate samples not shown within the main text figure. The triplicates shown in the original blots for Fig 5A were analyzed for the purpose of Fig 5B. (PDF) [file pbio.3001423.s016.pdf]
