# Supplementary material for: Assembly of infectious Kaposi’s sarcoma-associated herpesvirus progeny requires formation of a pORF19 pentamer
Source: PLoS Biol. 2021 Nov 4;19(11):e3001423. doi: 10.1371/journal.pbio.3001423 (PMC8568140; doi:10.1371/journal.pbio.3001423)
Supplement: Attachment — Submitted filename: Response_to_Reviewers.pdf [file pbio.3001423.s017.pdf]

Luebeck, July 6, 2021

Rebuttal letter

We thank both reviewers for their constructive comments and their positive feedback, for which we provide a point-by-point response below:

Reviewer #1: The article by Naniima et al. describes not only the crystal structure of pentameric pORF19 of KSHV (and its β-herpesviral ortholog) but it also integrates a series of mutagenesis, cellular, fluorescence and TEM microscopy experiments that shed light on the portal cap structure and its functional role in the herpesvirus life cycle. The findings are of interest and novel to the virology community.
Overall, the manuscript is a well presented, consistent piece of work and should be published in PLoS Biology after the suggested revisions are considered and properly addressed.

We thank the reviewer for the positive feedback.

INTRODUCTION
Page 1, 8 lines from top: why 'pseudo-icosahedral capsid'? better to use 'icosahedral capsid'

Due to the presence of the portal in the capsid and the fact that recent asymmetric herpesviral capsid reconstructions revealed asymmetric features within the KSHV capsid (Gong et al., 2019) we think that referring to a 'pseudo-icosahedral capsid' is less misleading.

Page 2: the description of the penton components would benefit from a schematic picture for those who are not expert in the herpesvirus field, serving as road map of the different CVSC (or CAT), triplex proteins etc. on top of a schematic icosahedron. It can be a schematic figure as supplementary Fig. S1 (this would also help the interpretability of Figure 3 in the context of the virion structure).

We thank the reviewer for this idea – we have prepared such a schematic figure and appended this as new Fig. S1 to the revised manuscript.

RESULTS:
Page 6, 2 lines from below: the sentence 'These data suggest…..significance' should be omitted because it does not represent an experimental result but the possible interpretative scenarios of the Figure S2. For this reason, it is better to move it to the Discussion section.

The sentence has been entirely removed from the manuscript as it does not contribute much to the Discussion section.

Page 7, 11 lines from below: please remove the second decimal to 19.22 kcal /mol also because the symbol '~' is used.

The second decimal has been removed.

Page 8: The text referring to Figure 3 and Figure 3 is confusing. Figure 3 labels are incorrectly placed; moreover Figure 3 can definitely be improved in terms of visual clarity by linking different regions with larger insets. Please clarify the main-text, the Figure 3 and its corresponding caption.

We apologize for the mislabeling of the old Fig. 3. We have now prepared a new Figure 3 that is colored according to the new Fig. S1 (see above), thereby supporting figure clarity. We have also revised the main text and the figure legend accordingly.

Page 11, 4 lines from top: the sentence 'negative-stain EM of pORF19KCTDCC demonstrated a 5-fold symmetry' is overstated. To demonstrate that the protein oligomerize forming a pentamer and thus displaying a 5-fold symmetry, a 2D classification would be more appropriated - and it is indeed recommended. Using negative-stain technique the Authors would not require a large set of images to prove their statement.
Figure 4H, as it is now, is not convincing and it does not show the existence of a homogeneous population of pORF19KCTDCC that forms a stable pentamer.

We thank the reviewer for this suggestion, we have now performed an SPA-analysis on the negative stain images. 2D class averages of approximately 12.000 particles revealed top and side views of the pentameric pORF19$_{KCTD}$CC and the top views clearly demonstrate the 5-fold symmetry of the molecule. These top and side views have been appended to the modified Figure 4 and an overview of the class averages is shown as new supplementary figure (Fig. S4).

DISCUSSION:
Page 19: 12 lines from top: the sentence 'The recombinant portal cap…..capsid disassembly'. It is not clear why the Authors mention this as it seems more of a plan that will possibly happen in the future. It does not add much to the discussion.

We agree with the reviewer that this sentence is not essential for the discussion and we have therefore removed it from the manuscript.

FIGURES:
Figure 3: see above comments
Figure 4H: see above comments

We thank the reviewer for the feedback, which we have addressed above.

Figure 7 caption: where is it the scale bar? Also, are these Z sections or stacked images?

We apologize for this technical issue – the scale bar is now visible in the top line of the fluorescent images. The images are stacked images and the individual panels were generated with ImageJ, and this information has now been appended to the figure legend.

METHODOLOGY:
Page 24: 'Structure analysis': The Author should state why the simulated map from the crystal structure was set to 5 Å resolution for the fitting into the portal density. The claimed resolution in the EMD-20430 is 7.6 Å for the asymmetric 3D reconstruction and it is likely that the resolution of the portal cap is slightly lower so why has the Author filtered the simulated map at 5 Å and not 7.6 or 8 Å?

As we have re-prepared figure 3 (see above), we also repeated the fitting procedure and for this purpose we fitted the monomeric and pentameric forms using a "SegFit" rotational search in UCSF Chimera into the asymmetric (C1) KSHV portal vertex reconstruction (EMD 20431). As the claimed resolution in EMD 20431 is 5.2 Å, but the resolution in the portal cap region of this map is likely to be considerably lower, we filtered the simulated map at 7.6 Å as suggested.

The authors didn't submit the PDB validation reports for their X-ray structures. In this case Table S2 is pretty convincing but it would a good practice for authors describing X-ray structures to include in their submission the corresponding PDB validation report: https://www.wwpdb.org/validation/validation-reports

All three validation reports are now added as accompanying items.

Reviewer #2: Summary

Herpesviral capsids are complex icosahedral assemblies composed of many copies of several viral proteins. One of these, the outer capsid protein conserved among all known herpesviruses - termed UL25 in HSV-1 - reinforces the capsid to allow it to withstand the pressure of the packaged genome and has also been implicated in genome packaging, cleavage, and retention. Prior to this study, the structural information has only been available for the HSV-1 UL25 homolog. Here, Naniima et al present the structures of three additional homologs of UL25: KSHV pORF19, MHV68 pORF19, and HCMV UL77. Interestingly, the KSHV pORF19 crystallized as a pentamer. By fitting the pentameric crystal assembly into the cryo-EM densities for the portal cap on the KSHV capsid, the authors conclude that the pORF19 pentamer observed in the crystals represents the portal cap. Mutations at the pentameric interface reduce viral titer and abolish capsid assembly, prompting the conclusion that formation of the pORF19 pentamer is essential for capsid assembly. The structural data are solid, but there are concerns regarding the overinterpretation of the biochemical and the virological data, the inadequate description of experimental procedures, and the missing controls.

We thank the reviewer for the constructive feedback.

Major criticisms

1. Cryo-EM reconstructions of HSV-1 and KSHV capsids (Gong 2019 and Liu 2019) have

revealed the presence of the portal cap and suggested that it contains UL25. However, the presence of UL25 within the portal cap has not yet been shown conclusively. The authors reference both Newcomb 2001 and Trus 2004 in the introduction (refs 9 and 10) as studies that agree with these proposed density assignments in the EM studies, yet both references focus on the HSV-1 portal composed of UL6 and do not examine the presence or involvement of UL25 at the portal cap. To be able to conclude that pORF19, a UL25 homolog, is a component of the portal cap (and that the pentameric structure reported here could be important for its formation), the authors need to show that the portal, indeed, contains pORF19, e.g., by immunogold labeling, or, alternatively, show that the deletion of pORF19 eliminates the portal cap from the capsids. Short of this, the conclusion that the pentameric pORF19 structure presented here is present in the portal cap should be toned down throughout the manuscript (including the title and the abstract) and presented as a hypothesis rather than a conclusion.

While we agree with the reviewer that the presence of pORF19 in the KSHV portal cap has not been conclusively shown and therefore understand the reviewer's concern, we consider it difficult to address this issue by immunogold labeling studies to unambiguously show the presence of pORF19 in the portal cap. Such an endeavour will be problematic for two reasons: 1) the absence of a good labelling antibody and 2) the simultaneous presence of pORF19 in the penton vertices that will make it difficult to distinguish the different vertices. We have therefore toned down our conclusions and offer the notion that our pentamer represents the portal cap as a likely, but not yet proven hypothesis. In addition, we have clarified the referencing in the revised manuscript.

2. The observation that mutations at the pentameric interfaces in pORF19 abolish capsid assembly in infected cells contradict the author's own data that show capsid assembly even in the absence of pORF19 in insect cells expressing capsid proteins. A bigger concern is the fact that this finding contradicts a body of literature on capsid assembly in alphaherpesviruses, such as HSV-1 and PRV, that shows that A- and B-capsids can form in infected cells even in the absence of UL25 (McNab 1998, Stow 2001, Mettenleiter 2006, Baines 2014). For example, C-terminal truncations in HSV-1 UL25 do not preclude formation of A- or B-capsids (Baines 2014). Furthermore, KSHV capsids can form in vitro the absence of the portal (Grzesik 2017), a finding confirmed by the authors.

We respectfully disagree with this reviewer in this point. It is true that in a baculovirus overexpression system, KSHV capsids can form in the absence of pORF19, but such an experiment only poorly reflects the capsid assembly process *in vivo*. The massive protein overexpression observed during baculovirus infection facilitates assembly of regular capsids even in the absence of the portal as stated by this reviewer, which has not yet been described in infected cells. It appears reasonable that the available protein levels in the host cell can affect or determine KSHV capsid formation in insect cells and we have discussed this hypothesis in the revised manuscript (lines 406-412).

While we cannot exhaustively explain the observed difference to the alphaherpesvirus literature, we do not claim that such mutations in a putative pentameric pUL25 interface will inhibit HSV-1 capsid assembly and we agree with the reviewer that there likely are unanticipated differences between alpha- and gammaherpesviruses in this respect (see reviewer's comment below). Our pORF19 knockout mutant is truncated at a similar position upstream of the

globular domain as a knockout mutant described in HSV-1 for pUL25 (McNab et al., 1998), however, the observed phenotype is strikingly different with no capsid formation in KSHV vs. A- and B-capsid formation in HSV-1. These data clearly indicate functional differences between HSV-1 pUL25 and KSHV pORF19 and we have now included that in the Results section of the revised manuscript.

One potential explanation is that the mutant viruses contain additional, unintended mutations that disrupt capsid assembly. This interpretation is consistent with poor in-trans complementation with WT pORF19.

The sequences of the entire bacterial artificial chromosomes used in this study have been verified by Next Generation Sequencing as stated in the Results section and we can therefore exclude unintended mutations that disrupt capsid assembly. As we state in the Results section we interpret the incomplete trans-complementation as a result of the presence of mutant pORF19, which likely acts as dominant-negative competitor in pORF19 pentamerization (at the portal) or formation of the CVSC helical bundle (at the penton) due to residual interactions with wt pORF19.

Alternatively, UL25 homologs could have differing roles in capsid formation during infection such that whereas HSV-1 UL25 is not essential for capsid formation, its KSHV homolog pORF19 is. This finding would be very interesting but requires that the authors perform additional controls, namely, a positive control showing that capsids do not form in the absence of pORF19 as well as a negative control showing that mutations located away from the pentameric interface has no effect on capsid formation.

We thank the reviewer for this constructive comment and as stated above we agree with the reviewer in this point. We already show the pORF19 deletion experiment suggested by the reviewer: the phenotype resulting from the pORF19 knockout is lack of capsid formation (Figs. 6-8), which is in stark contrast to the phenotype observed by McNab and colleagues for the HSV-1 KUL25NS mutant. It will be interesting to analyse in more detail whether this phenotype reflects an actual functional difference or whether it is due to secondary effects (e.g., differences in protein expression levels). With respect to the requested negative control, we consider usage of such a mutant virus with ‘mutations located away from the pentameric interface’ difficult to interpret. In view of the poorly understood pORF19 functions, it will be problematic to confirm that a particular mutation does not accidentally also impair a yet unknown important pORF19 function.

Along the same lines, it is difficult to reconcile the observed differences in the mutant phenotypes observed in Fig. 6 vs. Figs. 7 and 8. For example, the DQ and VL mutants appear to have a similar defect in progeny production (Fig. 6). However, only DQ appeared to form capsids (Fig. 7), predominantly A- and B-capsids, whereas the VL mutant produced no capsids (as observed in Figs, 7 and 8). Proper controls are needed to resolve these discrepancies.

We understand that the figure in its submitted format did not allow to reconcile the observed differences in the mutant phenotypes. We have therefore modified the Y-axis in Fig. 6B from a logarithmic scale into a linear scale. This clearly illustrates the presence of a gradient in the impact of the specific mutation on production of infectious progeny (e.g., the DQ mutant produces considerably more infectious particles than VL and loop mutants, in line with the results shown in Fig. 7 and 8).

3. There is a concern about the experimental rigor because some experimental procedures are not described in sufficient detail and because a few pieces of data are not shown. First, authors must remedy the lack of experimental details throughout the entire manuscript. For example, the description of the procedure used to test capsid association of the pentameric interface mutants is scant. The authors need to describe their experimental procedure in sufficient detail to allow others to reproduce their experiments. Citing the original paper (Desai, 2017) is insufficient. As another example, there is no mentioning in the methods of how the cysteine mutants were oxidized. Second, it is essential that the authors show all of their control data. For example, the data for the KO mutant of pORF19 should be shown to establish the validity of the conclusions. The authors should also clarify what KO mutation is, i.e., which amino acid residues are deleted.

We apologize for accidentally not having included all experimental procedures. While the experimental procedure for the oxidation of the pORF19 cysteine mutant is already present in the manuscript (Generation of pentameric pORF19$_{KCTD}$CC), we have now appended also a detailed method description for the baculovirus capsid assembly assay.
Of note, the data for the KO mutant are already shown in Fig. 6+7, however, we do not consider it useful to also show the electron micrographs of this mutant, as no capsids can be seen on these images similar to those showing the KSHV-Bac16loop mutant (Fig. 8C+D) and this is clearly stated in the main text. The exact residue borders that have been used for generation of the KO mutant have been appended to the description that was already present in the Methods section.

4. Describing pORF19 as a portal cap protein ignores its other functions and is not sufficiently supported by the available data. The authors should discuss a substantial body of literature on the role of HSV-1 UL25 in capsid stability and DNA packaging, e.g., Snijder 2019 and Cockrell 2009, as it would permit comparisons and contrasts with gammaherpesviruses. The excessive description of capsid structure in the already lengthy introduction could be shortened to accommodate these changes.

As described above and pointed out by the reviewer, our data suggest functional differences between pUL25 and pORF19, whereas the structural similarity of the two C-terminal domains is striking. Since less is known about the functions of pORF19 itself than about the structure (rendering comparisons with pUL25 difficult), we believe that for clarity it is better to focus on protein structure rather than protein function in the introduction.

Additional concerns

5. The manuscript is missing both the page numbers and line numbers.

Both page numbers and line numbers have been added to the revised manuscript.

6. Figure 3 is confusing and needs to be revised to be more helpful to the reader. The authors are trying to show that the pORF19 pentamer does not fit into the penton CVSC/CATC densities, yet the cartoon models shown in Fig. 3D are not the pentameric form that was obtained in the crystals. It would be helpful to show the fit of the pORF19 pentamer into the portal cap (as already shown in Fig. 3B) side-by-side with its fit into the CVSC/CATC density and include measurements showing that the pentamer does not fit the CVSC/CATC pORF19 density (one can use different colors to indicate where pORF19 is in the CVSC/CATC). In addition, the labels on Fig. 3 are incorrect (Fig. 3AB shows the portal, but in the text is referred to as the CVSC/CATC).

As outlined in the response to a comment by reviewer #1 (see above) we have now prepared a new Figure 3 that is colored according to the schematic drawing of penton and portal vertex in Fig. S1, thereby supporting figure clarity. At the same time, we have also revised the main text and the figure legend accordingly to improve figure clarity.

7. The authors present three new structures of UL25 homologs yet do not compare/contrast them in any meaningful way in Figure 1. It would be helpful to demonstrate structural similarities and differences using structural alignments, either all four aligned together or pairwise. It would also be helpful to have the sequence alignments indicating experimentally derived secondary structure for all four homologs. Comparisons of the electrostatic surface potential should also be shown in the main figure, instead of the supplement.

We show in Table S4 DALI scores for the comparison of the four available structures, a widely accepted measure for the structural similarity across orthologs. However, we thank the reviewer for the excellent suggestion of preparing a structural alignment. We have now appended such a structural alignment as new supplementary figure S3.

8. The pORF19 pentamer forms a tunnel lined with positively charged residues. The authors should discuss whether this is a common feature among the homologs or is specific to KSHV pORF19. Do homology models of the pentamers for the other homologs also show a positively charged tunnel? If not, what implications does this have regarding its role?

We agree with the reviewer that this is a burning question. The structural alignment shows that only one of the positively charged arginine and lysine residues in the funnel area are conserved across herpesviruses, suggesting that other positively charged residues can take over in pORF19 orthologs. We unfortunately think that homology modelling of the pentameric interface is not sufficiently reliable to assess such a crucial question due to insertions and

deletions in the loop regions of the individual proteins.

9. Fig. 2 should show the measurements listed in the Results section.

Outer and inner diameter of the pentamer have now been added to Fig. 2 including the circles that have been used to measure them.

10. The sentence "Based on cryo-EM data and a pORF19 homology model a loop…" is confusing as written and should be rephrased.

We have rephrased the sentence and hope it is clearer in the revised manuscript.

11. The cysteine mutants (P137C and P461C) are labeled incorrectly in Fig. 4F (currently labeled as P16C).

We thank the reviewer for spotting this mistake. We have now correctly labelled the cysteine mutant in Fig. 4.

12. Figure 4 is hard to follow. For example, panel F is located between G and H. It would be helpful if the authors moved the SEC panels to the top of the figure, from right to left (SEC buffer, LiAc buffer, then mutant chromatograms). Directly underneath, in a second row, could be panels B, F, H (in the text it is currently 3F, G and H discussed before C, D, and E). The third row would be C, E, D (see comment below) with the mutant information.

We thank the reviewer for this suggestion. As we added an additional panel to the revised Fig. 4 (see above), we used this opportunity to re-organize the individual panels mostly following the reviewer's suggestions.

13. To help the reader, the three mutants - DQ, loop, and VL - should be listed in the same order both in the text and the figures.

The pORF19 mutants appear now in the same order both in the text and the figures.

14. In the Results section describing the association of ORF19 mutants to capsid vertices, the authors state "In all cases, density gradient centrifugation of cell lysates revealed a clear capsid band at the expected position." What position is being referred to? It would be helpful to indicate this in the figure legend or, better, show the gradients themselves.

We agree with the reviewer that this statement was insufficient. We have now appended the position of the band together with the method for the capsid assembly assay and referred to this methods section in the results part. As this assay has been previously reproduced and reported several times, we feel that

showing the gradient might distract the reader from the central message of the manuscript.

15. Molecular masses of proteins in Fig. 5 and 6 should be indicated.

Molecular masses have been appended in the respective figure legends.

16. Fig. 7 legend refers to scale bars, yet none are shown on the figure.

We apologize for this technical issue – the scale bar is now visible in the top line of the fluorescent images.

17. Primers use in cloning should be provided as a Supplemental Table, for transparency.

We have now appended an additional Table (Table S6 of the revised manuscript) that lists the primers used throughout the study.

18. The strain name of E. coli for the KSHV BAC is incorrect as listed. GS1783 corresponds to HSV-1 BAC.

We have used the empty bacterial strain that was generated for “en passant mutagenesis” (Tischer et al., Methods Mol Biol, 2010) to insert the KSHV BAC, similar to the description provided in Brulois et al., J Virol, 2012.

19. In the methods, FBS is defined once, but further in the text FCS is used and is not defined.

We have now harmonized the nomenclature and used FBS throughout the revised manuscript.
