# Supplementary material for: Assembly of infectious Kaposi’s sarcoma-associated herpesvirus progeny requires formation of a pORF19 pentamer
Source: PLoS Biol. 2021 Nov 4;19(11):e3001423. doi: 10.1371/journal.pbio.3001423 (PMC8568140; doi:10.1371/journal.pbio.3001423)
Supplement: Attachment — Submitted filename: Response_to_Reviewers_R2.doc [file pbio.3001423.s018.doc]

Luebeck, November 30, 2026

Rebuttal letter

We thank the reviewer for the constructive comments and feedback, for which we provide a point-by-point response below:

Reviewer #2:

1. There are two major findings in this manuscript. The first finding is that the pORF19 pentamer crystal structure fits into the density of the portal cap in the KSHV capsid cryoEM reconstruction, suggesting that the crystal structure determined herein represents the portal cap. The second finding is that the capsids do not assemble in the absence of pORF19 or when the pORF19 pentameric interfaces are mutated.

The former finding is interesting but is not yet supported by any biochemical or functional data. So, its biological relevance is yet unclear. Nonetheless, the authors focus the entirety of their discussion on how the pORF19 pentamer could form at the portal cap (lines 502-541) and how their data suggest there is likely a UL77 portal cap in HCMV (lines 491-501). Considering the unclear biological relevance of the pentameric structure reported here, the authors could tone down this section of the discussion.

We have toned down our discussion in this respect and highlighted that “…. our crystal structure of the pentameric globular domain of KSHV pORF19 at 2.6 Å resolution could now close this gap …” (i.e., gap in knowledge on the structure of the herpesviral portal cap). Nevertheless, we consider it valuable to discuss also the most likely implications of our finding and therefore maintained this part of the discussion.

In order to highlight the impact of the second finding (i.e., the lack of capsid assembly in absence of pORF19), we have inserted an additional paragraph into the discussion that illustrates the implications of this finding.

2. By contrast, the latter finding is more interesting (it is even reflected in the title) and, potentially, more biologically significant because while pORF19 appears to play an essential role in capsid assembly in gammaherpesviruses, the HSV-1 UL25 homolog is dispensable for capsid formation (McNab 1998, Stow 2001, Mettenleiter 2006, Baines 2014). If true, this may suggest major differences in the assembly mechanisms of herpesviruses from different subfamilies. However, a thoughtful discussion on how pORF19 contributes to assembly and how this compares to pORF19 homolog in HSV is still lacking despite this being a major, biologically significant claim of the manuscript that is highlighted in the title.

We thank the reviewer for underlining the impact of our findings. As mentioned above, we have now added another paragraph to the discussion that highlights the implications and introduces different ways, how pORF19 could contribute to capsid assembly. However, we feel that it is difficult to discuss this in great detail and in particular to compare the role of pORF19 with the role of HSV-1 pUL25, as functional studies investigating the role of pORF19 are missing to date.

3. It is important that the authors rule out the possibility that pORF19 contributes to capsid assembly indirectly, for example, by being important for capsid protein expression (see, for example, a study on the role of the small capsid protein in KSHV capsid assembly - Sathish and Yuan 2010). The authors show that in the absence of pORF19, less pORF26 (triplex) is detected, and suggest that this could be due to reduced pORF26 expression (lines 410-412) but stop short of testing it. Ideally, the expression of other capsid proteins, such as the major capsid protein, should also be tested. This is a relatively straightforward experiments that would support a major (albeit undersold) claim of the manuscript. This would boost the claim that pORF19 contributes to assembly directly. At the bare minimum, the authors should discuss different potential mechanisms by which pORF19 could contribute to assembly in the Discussion. Is it known that the anti-pORF26 antibody is specific to capsid-bound pORF26? Knowing this would help with the data interpretation.

We agree with the reviewer that the next important step will be to decipher the role of pORF19 in capsid assembly. To initiate such studies, we have now added another paragraph on potential activities of pORF19 during capsid assembly (see above).

Unfortunately, it is not known, whether the α-pORF26 antibody recognizes only capsid-bound protein.

4. The observation that mutations at the pentameric interfaces in pORF19 abolish capsid assembly in infected cells contradict the data that show capsid assembly even in the absence of pORF19 in insect cells expressing capsid proteins. The authors now hypothesize that capsid assembly in insect cells can occur even in the absence of pORF19 because the capsid proteins are present at much higher levels in the KSHV-infected cells. But this is mentioned only briefly (lines 429-430) and should be discussed further, especially considering that the in vitro HSV-1 capsid assembly system largely recapitulates what is seen in HSV-1-infected cells (reviewed in Homa and Brown 1997, Brown and Newcomb 2011, and Heming 2017). While the authors agree that "These data clearly indicate functional differences between HSV-1 pUL25 and KSHV pORF19…", these differences are not compared in enough detail throughout the text. What is the evidence that the capsid protein levels are, indeed, much higher in insect cell system compared to KSHV-infected cells?

We respectfully disagree with the reviewer that our observation on the abolished capsid assembly by mutant viruses in iSLK cells and the results of the capsid assembly assay in insect cells are contradictory. Baculovirus infection in insect cells can lead to expression at very high target protein levels (up to 50% of total cellular protein; O’Reilly et al., 1994 - this reference has also been added to the manuscript) and constitutes a very special situation in virology. This special situation is reflected in assembly of regular capsids even in the absence of the portal. Similar protein levels will be difficult to reach in a KSHV-infected cell and assembly of regular capsids in the absence of the portal has not yet been described in infected cells.

5. Fig 4D is still confusing. The authors do not explain why the Y axis was normalized nor how it is normalized. The authors should include pORF19KCTD in the same panel and indicate LiAC buffer. The difference between the oligomer of pORF19KCTD and pORF19KCTDCC ox should also be explained.

Normalization of the c(s) distributions in Fig. 4D to the same area under the curve allows data collected at significantly different protein concentrations to be plotted together. Because the concentration of pORF19_KCTD_CC ox was more than a factor of 11 lower than the concentrations of the other proteins, details of CC ox c(s) distribution would be difficult to discern in an unnormalized plot. This is now stated in the modified figure legend for Fig. 4D.

The difference observed between the oligomer of pORF19_KCTD_ (right panel in Fig. 4A) and pORF19_KCTD_CC ox is due to an incomplete oligomerization of the wt protein, and this is explained in the main results section.
